# A new PHQ-2 for Chinese adolescents: identifying core items of the PHQ-9 by network analysis

**DOI:** 10.1186/s13034-023-00559-1

**Published:** 2023-01-21

**Authors:** Kaixin Liang, Sitong Chen, Yue Zhao, Yizhen Ren, Zhanbing Ren, Xinli Chi

**Affiliations:** 1 School of Psychology, Shenzhen University, Shenzhen, 518061 China; 2 The Shenzhen Humanities & Social Sciences Key Research Bases of the Center for Mental Health, Shenzhen University, Shenzhen, 518061 China; 3 School of Physical Education and Sport, Shenzhen University, Shenzhen, 518061 China; 4 Beijing Key Laboratory of Applied Experimental Psychology, Faculty of Psychology, Beijing Normal University, Beijing, 100875 China

**Keywords:** PHQ-9, PHQ-2, Depression, Screening, Adolescents, Reliability, Validity, Scale

## Abstract

**Background:**

The importance of preventing and treating adolescent depression has been gradually recognized in Chinese society, especially in the context of the COVID-19 pandemic. Early screening is the first step. The Patient Health Questionnaire-9 (PHQ-9) is a leading scale in the field of depression screening. To improve screening efficiency in large-scale screening, an even shorten scale is desirable. The PHQ-2, which only included two items measuring anhedonia and depressed mood, is an ultra-form of the PHQ-9. However, emerging evidence suggests that there may be a better short form for the PHQ-9, especially for adolescents. Therefore, using two large samples of Chinese adolescents, this study aimed to identify the core items of the PHQ-9 and examine the short form consisting of core items.

**Methods:**

Surveys were conducted among primary and middle school students in two Chinese cities with different economic levels during the COVID-19 pandemic. Two gender-balanced samples aged 10 to 17 (*n*_Sample 1_ = 67281, *n*_Sample 2_ = 16726) were collected. Network analysis was used to identify the core items of the PHQ-9, which were extracted to combine a short version. Reliability, concurrent validity, and the receiver operating characteristic curve (ROC) of the short form were examined. Analyses were gender-stratified.

**Results:**

Network analysis identified fatigue and depressed mood as core items in the PHQ-9 among Chinese adolescents. Items measuring Fatigue and Mood were combined to be a new PHQ-2 (PHQ-2 N). The PHQ-2 N displayed satisfactory internal consistency and current validity. Taking the PHQ-9 as a reference, the PHQ-2 N showed higher ROC areas and better sensitivity and specificity than the PHQ-2. The optimal cutoff score for the PHQ-2 N was 2 or 3.

**Conclusions:**

Fatigue and depressed mood are the central symptoms of the depressive symptom network. The PHQ-2 N has satisfactory psychometric properties and can be used in rapid depression screening among Chinese adolescents.

**Supplementary Information:**

The online version contains supplementary material available at 10.1186/s13034-023-00559-1.

## Introduction

Depression has become the leading cause of disability and the major contributor to suicide around the world, thus posing a heavy health burden on society [1]. With an estimated prevalence of 25% [2], addressing depression as a public health priority is urgent. Adolescent depression deserves additional concerns since depression tends to have its onset in adolescence [3]. Given that early treatment remediates the long-term trajectory of depression, adolescence is an essential period for evaluating and intervening in depression. Recent research reported that the global prevalence of depression among adolescents is estimated to be more than 25% during the COVID-19 pandemic [4, 5]. Monitoring depression during adolescence to improve the early detection and intervention of depression has been recommended in many countries [6, 7]. Recently, China’s National Health Commission and Ministry of Education have also successively recommended incorporating depression screening into the content of students’ health examinations [8, 9]. Screening for depression is the cornerstone of early recognition, diagnosis, and management [10]. Carrying out universal depression screening among adolescents based on appropriate screening tools to ensure early detection and intervention has generally reached a consensus [11].

In depression screening, using questionnaires to detect potential depression by identifying individuals with scores above a cutoff threshold is a common practice. Of all the tools for measuring depression, the Patient Health Questionnaire-9 (PHQ-9) is the most popular screener at present [12]. Developed based on the Diagnostic and Statistical Manual of Mental Disorders-IV (DSM-IV), the PHQ-9 reflects nine symptoms of Major Depressive Disorder (MDD) [13]. The scale is responded to on a 4-point Likert scale (0 = not at all, 3 = nearly every day). The total score of PHQ-9 scores ranges from 0 to 27 by simply summing up item scores, with a higher total score indicating more severe depression. A score of 10 or higher is recommended as a reasonable cutoff for potential depression [14, 15]. Owing to its brevity, simple scoring method, satisfying psychometric properties, as well as clinical utility, the PHQ-9 has been translated into various languages and used widely worldwide [16]. It has also shown stable and favorable psychometric properties among Chinese adolescents [17–19]. Moreover, the PHQ-9 has been recommended by the National Health Commission in China to be used for screening for depression among medical and health institutions and schools since 2020 [9].

However, in situations emphasizing efficiency (e.g., busy clinical practice, large-scale epidemiological studies, studies where depression is a secondary outcome and not the focus of the investigation), measures shorter than the PHQ-9 are even more desirable. To cope with these situations, researchers proposed a short version of the PHQ-9, which consists of two items for evaluating anhedonia and depressed mood [20]. These two symptoms considered core MDD symptoms in DSM-5 were extracted from the PHQ-9 to form the PHQ-2. The PHQ-2 is usually used in a two-step procedure in which the full PHQ-9 scale or the remaining PHQ-9 items are only applied after a positive screening of the PHQ-2 [14, 21]. Incorporating such an ultra-short version with the PHQ-9 in large-scale depression screening may be a resource-efficient approach as it can greatly improve screening efficiency and reduce the burden on respondents.

Although some studies have validated the utility of the PHQ-2, items of the PHQ-2 may need to be reconsidered when the aim is to provide a primary measurement for depression screening among adolescents. Several reasons may justify the reconsideration. First of all, specifying anhedonia and depressed mood as ‘core symptoms’ was mainly based on clinical experience by observing adults seeking treatment or undergoing treatment, but the manifestation of depression symptoms in adolescents may be different from that in adults. For instance, by comparing the presentation of DSM-IV depression symptoms in adolescents and adults with MDD, researchers found that somatic symptoms (e.g., loss of energy, appetite change) were more common in adolescent MDD than in adult MDD, and loss of energy was associated with the highest probability of adolescent MDD [22]. However, the existing PHQ-2 does not include items reflecting somatic symptoms as both anhedonia and depressed mood belong to affective/cognitive aspects. Not assessing somatic symptoms like energy loss in adolescents may result in potential depression cases being missed. Besides, the screening ability of the PHQ-9 original algorithm, which emphasizes anhedonia and depressed mood, is unsatisfactory [23, 24]. Following the diagnosis criteria of DSM-IV, the PHQ-9 initially suggested the following algorithm: if five or more items score 2 or higher (more than half the days), and at least one item should include anhedonia or depressed mood, the presence of depression can be considered. Although this algorithm follows the rules of DSM-IV more closely, it fails to be more accurate than the simple addition scoring (summing up item scores) that is more commonly used currently [24]. This implies that the importance of at least one of the two items (anhedonia and depressed mood) may be overestimated, or the significance of other items may be underestimated.

Notably, by aggregating findings from network analysis in clinical and population studies, a recent systematic review found that fatigue and depressed mood were the most critical MDD symptoms across studies, with anhedonia being slightly less central in networks of MDD [25]. From the emerging perspective of network analysis, the mental disorder is conceptualized as a complex dynamic network composed of interacting symptoms [26, 27]. In other words, the connection between symptoms constitutes the disorder, not the symptom caused by the disorder. Different symptoms (called nodes in the network) own different importance to the network constituted. Nodes with more or stronger connections with other nodes are considered central nodes (or core nodes). Central nodes are presumed to play a more prominent role in the occurrence and development of mental disorders because the activation of central nodes might directly affect other nodes [27]. Therefore, items measuring core symptoms identified by network analysis maybe be more suitable to be used in depression screening as the presence of core symptoms implies a high risk of developing more severe depression. Additionally, studies have found that after the outbreak of COVID-19, the network structure of psychopathology symptoms changed to some extent [28–30], and node centrality of each symptom in the network might have altered. Consequently, updated data are needed to analyze the core symptoms of depression and provide a more cutting-edge reference as the pandemic continues. Collectively, emerging evidence suggests that there may be a better ultra-short form beyond the PHQ-2, at least for Chinese adolescents.

Against the above background, by analyzing data from Chinese adolescent samples, this study aimed to identify the core items of the PHQ-9 by network analysis and combine the core items into a new short version. The reliability, validity, cutoff, sensitivity, and specificity of the new short version were calculated and compared with the PHQ-2. The study would provide empirical evidence about the core items of the PHQ-9 and may provide a new ultra-short version of the PHQ-9 for rapid depression screening among Chinese adolescents.

## Methods

### Participants

This study used two separate samples of Chinese adolescents collected after the outbreak of COVID-19. Sample 1 was collected from a cross-sectional survey conducted in Shenzhen (an economically highly developed city in Guangdong, China) in March 2021, consisting of 67281 adolescents aged 10–17 years (mean age = 13.0, standard deviation [SD] = 1.8), including 34909 (51.9%) males and 32372 (48.1%) females. Sample 2 was collected from a cross-sectional survey conducted in Hechi (an economically developing city in Guangxi, China) in May 2020, consisted of 16726 adolescents aged 10–17 years (mean age = 14.2, SD = 1.8), including 7590 (45.4%) males and 9136 (54.6%) females. All participants were enrolled at local public primary and middle schools. We invited participants to fill out our online questionnaire via Wenjuanxing (a Chinese online questionnaire platform, https://www.wjx.cn/). Since the questionnaire could only be submitted after all questions were completed, there were no missing values in the samples. All participants gave informed consent before data collection. Both surveys to collect Sample 1 and Sample 2 were in collaboration with local bureau of education and parents of participants gave informed consent to the investigation. The Human Research Ethics Committee of the corresponding author’s affiliated institution approved the studies generating the data used in study (Code number: 2020005).

### Measures

The PHQ-9 evaluates the frequency of depression symptoms in the past 2 weeks. Items include (1) Little interest or pleasure in doing things (Anhedonia); (2) Feeling down, depressed, or hopeless (Mood); (3) Trouble falling or staying asleep, or sleeping too much (Sleep); (4) Feeling tired or having little energy (Fatigue); (5) Poor appetite or overeating (Appetite); (6) Feeling bad about yourself, or that you are a failure or have let yourself or your family down (Guilt); (7) Trouble concentration on things, such as reading the newspaper or watching television (Concentration); (8) Moving or speaking so slowly that other people could have noticed, or the opposite, being so fidgety or restless that you have been moving around a lot more than usual (Motor); (9) Thoughts that you would be better off dead or of hurting yourself in some way (Suicide). Each item is given a four-point rating (0 = not at all, 3 = nearly every day), and the total score of the PHQ-9 can range from 0 to 27. A score of 10 or higher has acceptable diagnostic properties for detecting major depression [15, 23]. In the current study, we used the Chinese version of the PHQ-9, which has been well-validated in Chinese populations, including adolescents [18, 31, 32].

To assess the criterion validity of the new short form of the PHQ-9, the Generalized Anxiety Disorder Scale-7 (GAD-7), the Internet Addiction Test (IAT), the Connor-Davidson Resilience Scale-10 (CD-RISC-10), and the 5Cs Positive Youth Development Scale-Very Short Form (PYD-VSF) were also measured. The GAD-7 is a commonly used questionnaire that assesses the frequency of anxiety symptoms over the past 2 weeks and has the same way of rating and scoring as the PHQ-9. The IAT asks participants about ten IA behaviors on a “Yes” or “No” checklist, and more behaviors indicated more severe internet addiction. The CD-RISC-10 measures the level of resilience on a 5-point Likert scale (0 = never, 4 = almost always), with higher total scores indicating higher levels of resilience. The PYD-VSF assesses positive development levels from five aspects, including competence, confidence, character, connection, and caring. The Chinese versions of the above scales have been validated in Chinese adolescents [33–36].

### Data analyses

Sample 1 and Sample 2 were split by gender. The following data analyses were carried out for subsamples respectively. Network analyses were performed to estimate the network structure consisting of depressive symptoms. In networks, observed variables are called nodes, and estimated relations between nodes are called edges. The network model included all items from the PHQ-9, thus resulting in nine nodes. Following the tutorial on Network Psychometrics with R [37], we estimated the network using a Gaussian Graphical Model (GGM), which presents partial correlations between nodes. Considering the item scores of depressive symptoms were not normally distributed, the Spearman correlation was selected. As the sample size was large, we adopted the ggmModSelect algorithm (tuning parameter = 0.5). Stronger correlations between nodes are presented by thicker edges. The accuracy and stability of edge estimates were assessed using nonparametric bootstrapping (*n* = 1000). To identify the most important or central nodes in the network, strength centrality was calculated to estimate the centrality of each item [38, 39]. Strength centrality estimates how strongly a node is directly connected with the network. Considering that the network was estimated from data and may be subject to sampling variation, we use case-drop bootstrapping (*n* = 1000) to assess the accuracy and stability of strength centrality estimates. To ensure interpretable differences in strength centrality, we used the nonparametric bootstrapped difference test (*n* = 1000) to examine whether there was a significant difference between the strength centrality of the two nodes. Items with the highest node strength would be considered core items and combined to form the short version of the PHQ-9. Results are presented following the reporting standards for psychological network analyses in cross-sectional data [40].

Then, we calculated the mean score and standard deviation (SD) of each item and scale. The independent sample *t*-test was conducted to compare the scores between genders. The effect size of the difference was indicated by Cohen’s *d*. Three reliability estimators (i.e., McDonald’s ω, Cronbach’s α, and Greatest Lower Bound) were applied to measure the internal consistency reliability of the new short form and PHQ-2. Regarding criterion validity, Spearman correlations between the short form and the PHQ-9, the GAD-7, the IAT, the CD-RISC-10, and the PYD-VSF were calculated. Sensitivity and specificity were determined by receiver operating characteristic (ROC) analysis with the PHQ-9 (≥ 10) as the reference. The area under the curve (AUC) and 95% CI presented the overall accuracy of the new short-form and PHQ-2 relative to PHQ-9. The optimal cut-off scores for the short-form and the PHQ-2 were determined by the largest Youden index (sensitivity + specificity−1), which indicates a balance between sensitivity and specificity [41]. Finally, to provide normative data on PHQ scales, Sample 1 and Sample 2 were combined to obtain a more representative sample. Normative data for the PHQ-9, PHQ-2, and the new short-form was generated by calculating gender-specific percentages for each scale.

Network analyses were conducted in RStudio (version 2022.07.2), ROC analyses were conducted in MedCalc (version 20.022), and other analyses were conducted in SPSS (version 27). The significance level for all analyses was set at *p* < 0.05.

## Results

### Identifying the core items

**Fig. 1:**
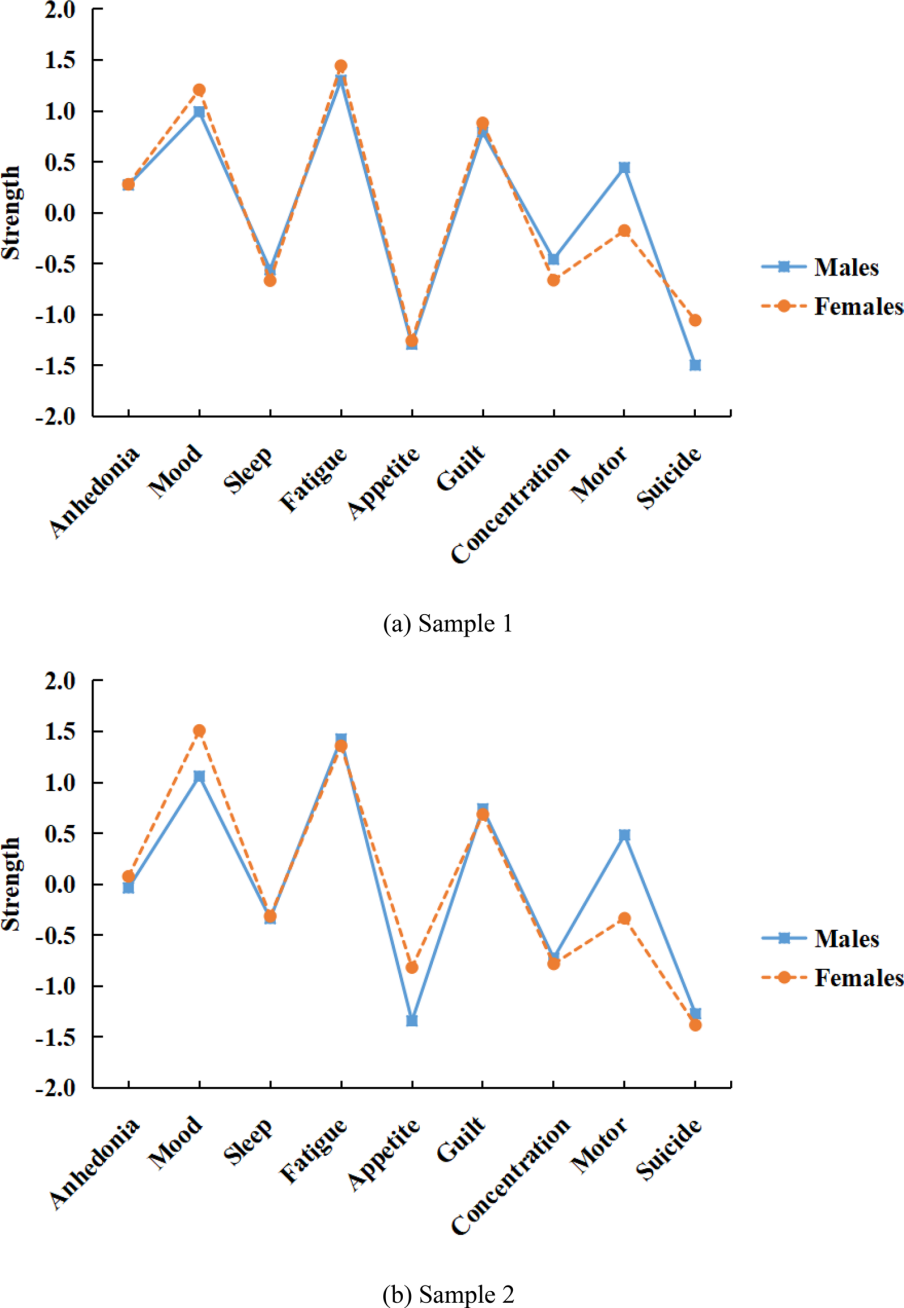
Node strength centrality of PHQ-9 items in the network. Note. Values of node strength centrality are normalized

**Fig. 2:**
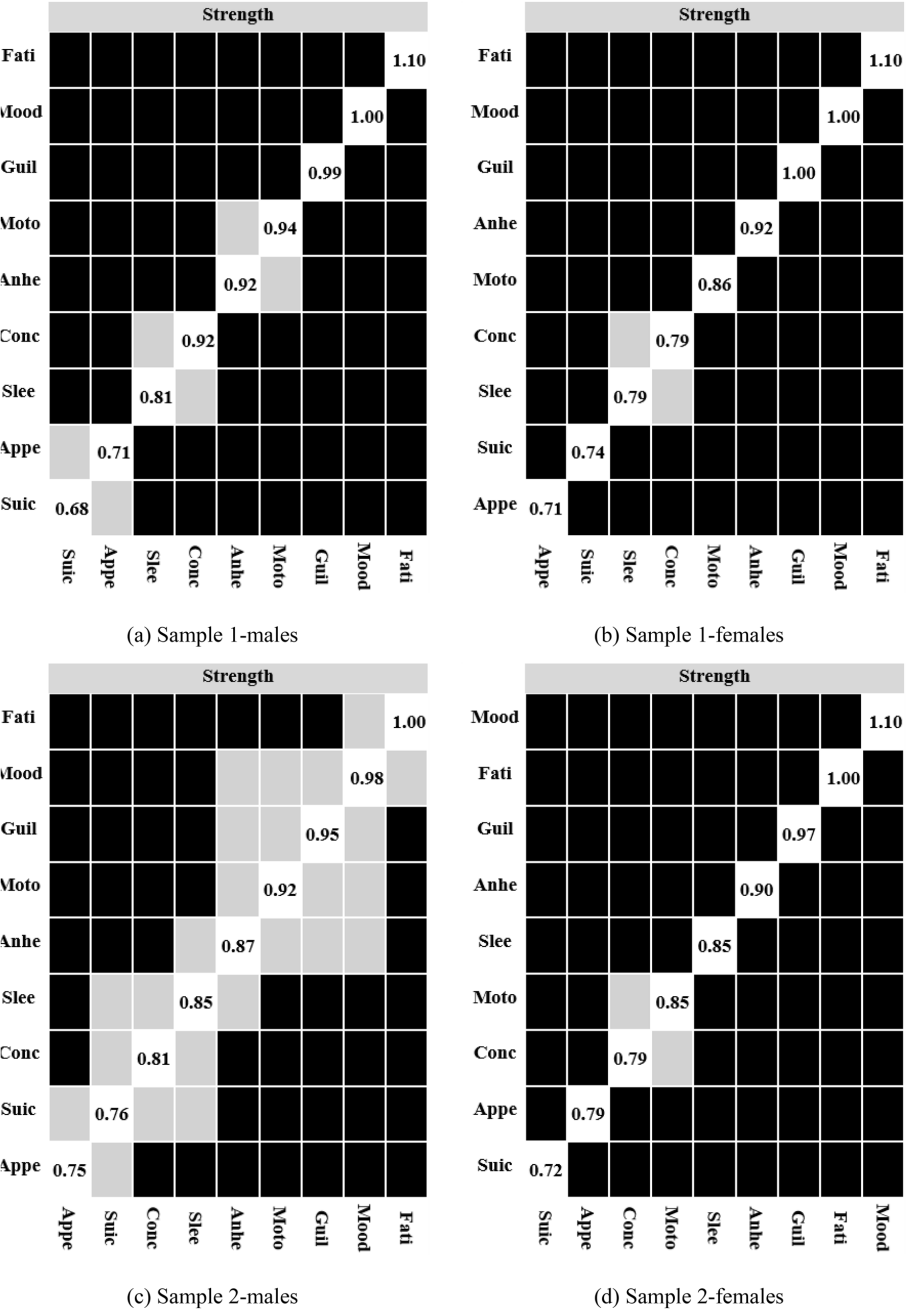
Bootstrapped difference test for node strengths. Note. The figure shows per centrality measures the difference test (with an alpha of 0.05) between the estimated and bootstrapped node strength. Black boxes indicate that node strength differ significantly, while gray boxes indicate no significant difference. The numbers in the white boxes refer to the raw value of the node strength

Visualized networks are presented in Additional file 1: Figure S1. In general, edge estimates were accurate and reliable (Additional file 1: Figure S2). Figure 1 displays the strength centrality of each PHQ-9 item. Fatigue and Mood showed the highest strength centrality in both gender-specific networks of Sample 1 and Sample 2. Results of difference tests showed that Fatigue and Mood were significantly more central than most other items (Fig. 2). Besides, strength centrality estimates were stable, with CS coefficients of 0.75 in all subsamples, indicating that 75% of the data could be dropped to retain 95% certainty with a correlation of 0.7 with the original data set. Therefore, items measuring depressed mood and fatigue were identified as the two core items in the PHQ-9 and formed the new short form, named the PHQ-2 N.

### Descriptive statistics of item and scale scores

As shown in Table 1, females reported significantly higher item scores than males in both Sample 1 and Sample 2 (Cohen’s *d* ranged from 0.04 to 0.29, all *p* ≤ 0.001). Consistently, females got significantly higher scores on the PHQ-9, the PHQ-2, and the PHQ-2 N in all sub-samples (Cohen’s *d* ranged from 0.21 to 0.28, all *p* ≤ 0.001).

**Table 1:** Descriptive statistics of PHQ items and scales

	Sample 1				Sample 2			
	Male (n = 34909)	Female (n = 32372)	Cohen’s *d*	*p*	Male (n = 7590)	Female (n = 9136)	Cohen’s *d*	*p*
	Mean (SD)	Mean (SD)			Mean (SD)	Mean (SD)		
Item score								
Anhedonia	0.67 (0.75)	0.79 (0.77)	0.16	< 0.001	0.60 (0.80)	0.77 (0.83)	0.21	< 0.001
Mood	0.54 (0.73)	0.72 (0.80)	0.24	< 0.001	0.43 (0.72)	0.65 (0.82)	0.29	< 0.001
Sleep	0.47 (0.77)	0.60 (0.85)	0.16	< 0.001	0.53 (0.84)	0.72 (0.92)	0.21	< 0.001
Fatigue	0.59 (0.78)	0.74 (0.83)	0.19	< 0.001	0.51 (0.77)	0.69 (0.84)	0.22	< 0.001
Appetite	0.50 (0.76)	0.60 (0.82)	0.13	< 0.001	0.39 (0.72)	0.56 (0.83)	0.22	< 0.001
Guilt	0.56 (0.82)	0.75 (0.91)	0.22	< 0.001	0.55 (0.83)	0.83 (0.96)	0.31	< 0.001
Concentration	0.44 (0.72)	0.53 (0.77)	0.13	< 0.001	0.43 (0.76)	0.54 (0.81)	0.14	< 0.001
Motor	0.40 (0.72)	0.43 (0.75)	0.04	< 0.001	0.36 (0.70)	0.39 (0.72)	0.05	0.001
Suicide	0.29 (0.65)	0.43 (0.77)	0.20	< 0.001	0.22 (0.58)	0.36 (0.71)	0.20	< 0.001
PHQ-9 total score	4.46 (5.60)	5.60 (5.60)	0.21	< 0.001	4.02 (4.98)	5.51 (5.65)	0.28	< 0.001
PHQ-2 N total score	0.94 (1.32)	1.34 (1.49)	0.24	< 0.001	0.93 (1.32)	1.34 (1.49)	0.28	< 0.001
PHQ-2 total score	1.03 (1.33)	1.42 (1.47)	0.22	< 0.001	1.03 (1.33)	1.42 (1.47)	0.28	< 0.001

### Reliability and validity of the PHQ-2 N and PHQ-2

As listed in Table 2, in all sub-samples, internal consistency estimates of PHQ-2 N were larger than 0.718 and that of PHQ-2 were larger than 0.703. Scores of both scales were positively correlated with scores of the PHQ-9 (PHQ-2 N: *r* ranged from 0.85 to 0.89, PHQ-2: *r* ranged from 0.86 to 0.89), GAD-7 (PHQ-2 N: *r* ranged from 0.61 to 0.74, PHQ-2: *r* ranged from 0.60 to 0.73), and IAT (PHQ-2 N: *r* range from 0.35 to 0.45, PHQ-2: *r* ranged from 0.36 to 0.44); conversely, scores of both scales were negatively correlated with scores of CD-RISC-10 (PHQ-2 N: *r* range from −0.38 to −0.14, PHQ-2: *r* ranged from −0.39 to −0.14) and PYD-VSF (PHQ-2 N: *r* range from −0.47 to −0.28, PHQ-2: *r* range from −0.47 to −0.29).

**Table 2:** Reliability and validity of the PHQ-2 N and PHQ-2

	Sample 1						Sample 2					
	Male			Female			Male			Female		
	PHQ-9	PHQ-2 N	PHQ-2	PHQ-9	PHQ-2 N	PHQ-2	PHQ-9	PHQ-2 N	PHQ-2	PHQ-9	PHQ-2 N	PHQ-2
Internal consistency												
McDonald’s ω	0.911	0.797	0.802	0.915	0.804	0.802	0.897	0.719	0.703	0.908	0.757	0.737
Cronbach’s α	0.909	0.797	0.802	0.914	0.803	0.802	0.896	0.718	0.704	0.907	0.758	0.738
Greatest Lower Bound	0.931	0.797	0.802	0.935	0.803	0.802	0.915	0.718	0.704	0.928	0.758	0.738
Criterion validity (correlations with criterion measures)												
PHQ-9	/	0.876	0.880	/	0.892	0.884	/	0.849	0.859	/	0.886	0.885
GAD-7 (anxiety)	0.765	0.696	0.678	0.810	0.744	0.725	0.681	0.606	0.601	0.739	0.672	0.659
IAT (internet addiction)	0.424	0.359	0.366	0.483	0.425	0.422	0.402	0.351	0.359	0.490	0.449	0.441
CD-RISC-10 (resilience)	−0.329	−0.292	−0.303	−0.42	−0.379	−0.388	−0.162	−0.138	−0.144	−0.284	−0.254	−0.255
PYD-VSF (positive youth development)	−0.424	−0.379	−0.382	−0.521	−0.472	−0.471	−0.323	−0.278	−0.288	−0.415	−0.375	−0.381

### Comparing the sensitivity and specificity between the PHQ-2 N and PHQ-2

**Fig. 3:**
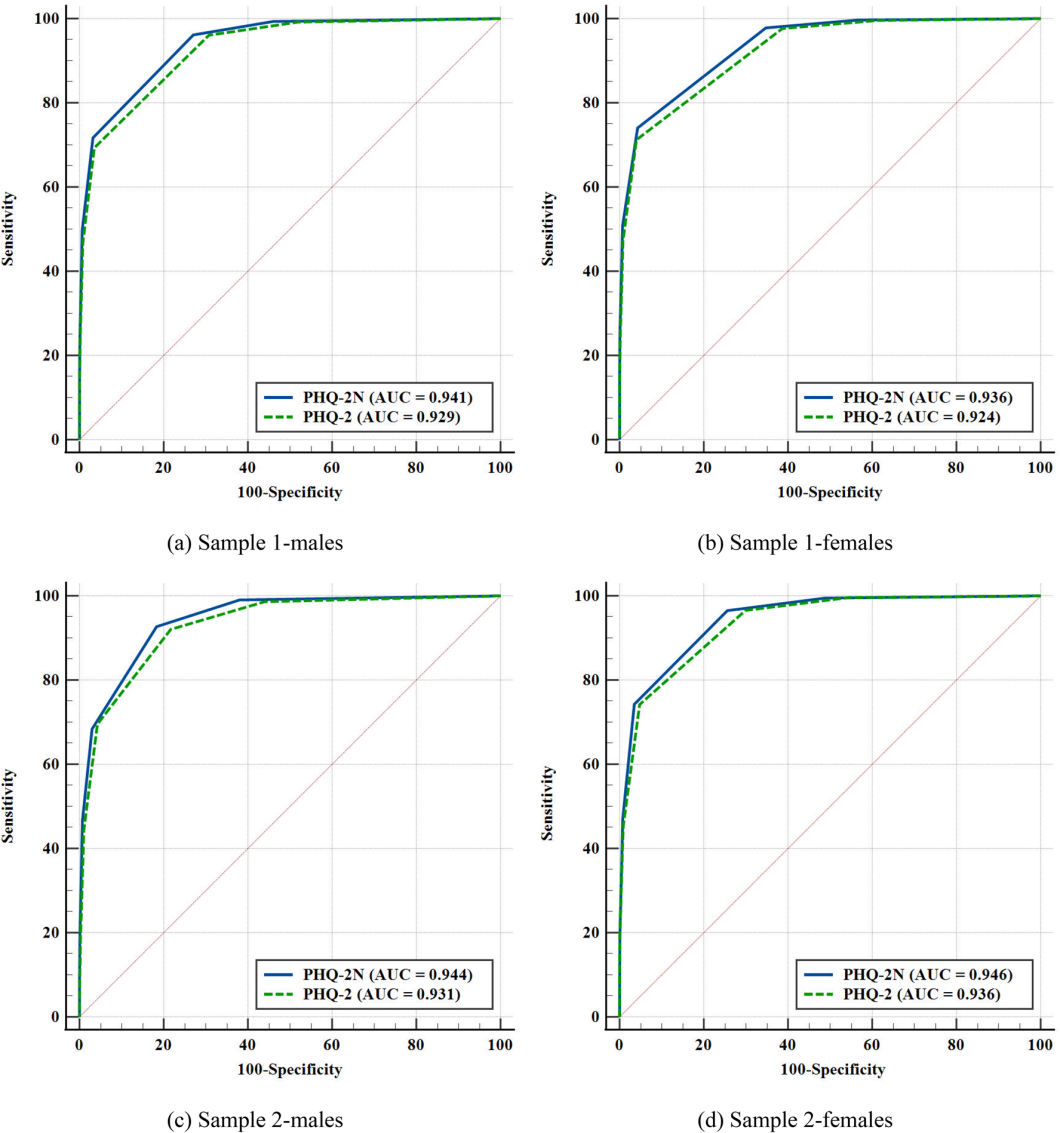
Receiver operating characteristic curve of the PHQ-2 N and PHQ-2

As shown in Fig. 3, with PHQ-9 ≥ 10 as the reference, the PHQ-2 N performed better than the PHQ-2 with significantly higher estimates of AUC in all sub-samples (all *p* < 0.001). The sensitivity and specificity of the PHQ-2 N and PHQ-2 are presented in Table 3. The Yonden index suggested that for PHQ-2 N and PHQ-2, a score of 2 or 3 would be the appropriate cutoff. Adopting the same cutoff, the PHQ-2 N had a higher Youden index than the PHQ-2 with better sensitivity or specificity. Normative data of the PHQ-2 N and PHQ-2 are presented in Table 4 (normative data of the PHQ-9 can be found in Additional file 1: Table S1). With the cutoff set at 2, the PHQ-2 N screened 35.4% of males and 45.2% of females with PHQ-9 scores higher than 10, and the PHQ-2 screened 38.6% and 45.4%. With the cutoff set at 3, the PHQ-2 N screened 11.8% males and 17.5% females, and the PHQ-2 screened 12.0% and 17.2%.

**Table 3:** Sensitivity and specificity of the PHQ-2 N and PHQ-2

	Male				Female			
	Cutoff	Sensitivity	Specificity	YI	Cutoff	Sensitivity	Specificity	YI
Sample 1								
PHQ-2 N	1	0.99	0.54	0.53	1	1.00	0.44	0.43
	**2**	**0.96**	**0.73**	**0.69**	2	0.98	0.65	0.63
	**3**	**0.72**	**0.97**	**0.69**	**3**	**0.74**	**0.96**	**0.70**
	4	0.50	0.99	0.49	4	0.51	0.99	0.50
	5	0.23	1.00	0.23	5	0.25	1.00	0.25
PHQ-2	1	0.99	0.48	0.47	1	1.00	0.39	0.38
	2	0.96	0.69	0.65	2	0.98	0.61	0.59
	**3**	**0.69**	**0.97**	**0.66**	**3**	**0.71**	**0.96**	**0.67**
	4	0.47	0.99	0.46	4	0.47	0.99	0.47
	5	0.22	1.00	0.22	5	0.23	1.00	0.23
Sample 2								
PHQ-2 N	1	0.91	0.62	0.61	1	1.00	0.51	0.51
	**2**	**0.93**	**0.82**	**0.74**	**2**	**0.97**	**0.74**	**0.71**
	3	0.68	0.97	0.65	**3**	**0.74**	**0.97**	**0.71**
	4	0.47	0.99	0.46	4	0.47	0.99	0.46
	5	0.18	1.00	0.18	5	0.19	1.00	0.19
PHQ-2	1	0.99	0.56	0.55	1	1.00	0.46	0.45
	**2**	**0.92**	**0.78**	**0.70**	2	0.97	0.70	0.67
	3	0.70	0.96	0.65	**3**	**0.74**	**0.95**	**0.69**
	4	0.44	0.99	0.43	4	0.45	0.99	0.44
	5	0.19	1.00	0.19	5	0.20	1.00	0.20

*YI* Yonden index. The cutoff values corresponding to the maximized Youden index are bold

**Table 4:** Normative data of the PHQ-2 N and PHQ-2

PHQ-2 N					PHQ-2				
Total score	Male		Female		Total score	Male		Female	
	Percentage	CP	Percentage	CP		Percentage	CP	Percentage	CP
0	48.4	48.4	36.6	36.6	0	43.3	43.3	32.6	32.6
1	17.2	65.6	18.3	54.8	1	19.1	62.4	19.1	51.6
2	22.6	88.2	27.7	82.5	2	25.5	88	31.2	82.8
3	5.0	93.2	7.4	89.8	3	5.4	93.4	7.5	90.4
4	4.0	97.2	5.6	95.4	4	3.8	97.2	5.3	95.7
5	1.0	98.2	2.0	97.4	5	1.0	98.2	1.9	97.6
6	1.8	100	2.6	100	6	1.8	100	2.4	100

Data are shown in percentages (%), *CP* cumulative percentage

## Discussion

Using two separate data sources obtained from Chinese adolescents in two cities with different economic levels, we identified fatigue and depressed mood were two core items of the PHQ-9. The two items were combined to form the PHQ-2 N. The PHQ-2 N displayed satisfactory internal consistency reliability and criterion validity. With the PHQ-9 as the reference, the PHQ-2 N displayed better sensitivity and/or specificity than the PHQ-2. A score of 2 or 3 would be the optimal cutoff for the PHQ-2 N.

Based on node strength from network analysis, we identified depressed mood and fatigue as the core items. Despite differences in PHQ scores between males and females, the network analysis yielded similar results for both genders. The results of the present study support the results of previous network analyses that also used the PHQ-9 to measure depression in adolescents [42, 43]. Notably, the finding seems not limited to adolescent samples. A systematic review synthesizing results from network analyses of depression symptoms [25] highlighted the critical role of depressed mood and fatigue. Additionally, findings from a recent randomized clinical trial (mean age of participants was 40.18) also suggested that depressed mood and fatigue seemed to be the most central MDD symptoms and thus may be viable targets for antidepressant interventions [26]. Network analysis tests connections between symptoms, and symptoms closely connected to other symptoms are regarded as central symptoms. Central depression symptoms like depressed mood and fatigue are assumed to have a widespread impact on the development of depression (which often occurs in adolescence or early adulthood) because their activation may trigger other symptoms. Although more studies are needed to determine the root cause symptom (symptom that first appear and activate other symptoms), this study, along with previous findings from network analysis suggests that depressed mood and fatigue are at the core of the network of depression symptoms and adolescents scored higher at these two symptoms would face a higher risk of depression. Hence, within the scope of developing a prescreen scale for depression screening among adolescents, assessing depressed mood and fatigue may be particularly important.

Moreover, the PHQ-2 N can measure more comprehensive content than the PHQ-2. MDD symptoms are reflected in affective, cognitive, and somatic aspects [44]. Individuals diagnosed with MDD may have different profiles of symptoms [22, 45]. For example, phenotypic heterogeneity has been recognized in the manifestation of depression symptomatology in adults and adolescents and fatigue was more likely to be endorsed as a symptom in adolescents [22]. Correspondingly, specific symptoms measured by the PHQ-9 can also be divided into cognitive-affective and somatic dimensions [46–48]. Both depressed mood and anhedonia are consistently regarded as belonging to the cognitive-affective dimension while fatigue pertains to the somatic dimension across studies [46, 49, 50]. Hence, compared to the PHQ-2 with only cognitive/affective items, an ultra-short form such as the PHQ-2 N involving both cognitive/affective- and somatic-related symptoms is more comprehensive and may be more suitable in screening adolescent depression.

In addition, with the PHQ-9 as the reference, the PHQ-2 N displayed more advanced sensitivity and specificity. In other words, compared with the PHQ-2, the PHQ-2 N had a lower proportion of false positives and false negatives and thereby had a better screening ability in distinguishing between depressed and non-depressed adolescents. This adds evidence to the importance of measuring fatigue and depressed mood as discussed above. The PHQ-2 N would detect more cases (PHQ-9 ≥ 10) and avoid more false positives. As shown in Table 4, relative to the PHQ-2, the PHQ-2 N screened fewer positive screens and thus requires fewer adolescents to undergo the full PHQ-9 or other treatment with the cutoff being 2 or 3, reducing the burden of respondents involved in the screening. The results support that the PHQ-2 N may be a better ultra-short version than the PHQ-2.

In line with previous studies examining the optimal cutoff of the PHQ-2 [14, 20], the current study suggested that the PHQ-2 N had balanced sensitivity and specificity at the cutoff score of 2 and 3. Sensitivity and specificity differ upon the threshold score of 2 and 3. As the cut-point increased, specificity improved at the expense of reduced sensitivity inevitably (Table 3). Therefore, the cutoff should be further determined according to the purpose of use. Specifically, if the goal is to improve the detection rate as much as possible, 2 points would be prudent and more certain that all those with a PHQ-9 total score meeting the threshold are detected.

Some strengths and implications of this study are worth mentioning. First of all, we used two independent samples consisting of adolescents in cities with different economic levels which strengthens the robustness of the results. Second, the sample size of both samples was large and the gender distribution was balanced, which allowed us to conduct gender-stratified analyses to take gender differences in depression into account. Third, we have generated normative data for the three PHQ scales, as our data were collected after the COVID-19 pandemic, which had a negative impact on adolescents’ mental health and led to increased depression [51], along with the consideration that the pandemic is still ongoing, our normative data of PHQ scales can offer a more up-to-date reference. Fourth, all measures used in the study have been tested for reliability and validity. As far as the authors can determine, this study is the first to achieve the goal of abbreviating the PHQ-9 through the statistical procedure. Although our samples include only Chinese adolescents, we did provide a simple and effective screening tool (PHQ-2 N) for rapid and large-scale depression screening in Chinese adolescents.

This study is exploratory in nature and there are limitations that need to be addressed in future studies. First of all, since the primary purpose of this study was to establish a preliminary screening scale, this study included only general adolescents and lacked diagnostic measures to evaluate the criterion validity of the PHQ-2 N. Consequently, the findings of the current study may not be generalizable to the clinical population. Although a systematic review of depression networks suggested that the sample type (clinical vs. population-based settings) did not confound the result that fatigue and depressed mood are the most central symptoms [25], future studies are encouraged to add diagnostic gold standards in adolescent samples to verify or modify the findings of this study. Moreover, this study only analyzed data from adolescent respondents recruited from two Chinese cities, and it is unclear whether the findings can be generalized to other samples of adolescents or even adults in other countries. Considering the same item may display different nuances depending on translation, which can lead to different interpretations of the symptom content across different cultures and contexts, we suggest future studies examine the psychometric properties of the PHQ-2 N in a wider range of populations and areas to confirm or refute the findings. Given the PHQ-2 has more published evidence of its reliability and validity, further research comparing the PHQ-2 and PHQ-2 N is warranted.

## Conclusions

This study suggests that depressed mood and fatigue might be the core symptoms among Chinese adolescents. The PHQ-2 N measuring depressed mood and fatigue showed satisfactory psychometric properties, including better sensitivity and specificity than the existing PHQ-2. The PHQ-2 N is a promising ultra-short tool for depression screening in Chinese adolescents, and the recommended cutoff score is 2 or 3.

## Supplementary Information

**Additional file 1: Figure S1.** Network structure of PHQ-9 items. Note. The stronger the association between nodes, the thicker and more saturated the edge is represented in the network. Blue edges represent positive associations. **Figure S2.** Accuracy of edge weights. Note. The gray area shows the bootstrapped confidence intervals of the estimated edge weights for the estimated network. The red values (connected by the red line) indicate the sample mean values for the bootstrapped edge weights. The black values indicate the estimated edge weights. **Figure S3.** Stability of nose strength centrality. Note. The plot shows the average correlation between the strength for the estimated network and the bootstrapped network. The lines indicates the mean correlation between centrality measures and the area around the indicates the 2.5th till the 97.5th quartile. **Table S1.** Normative data of the PHQ-9.

## Data Availability

The datasets used and/or analyzed during the current study are available from the corresponding author on reasonable request.

## Abbreviations

PHQ-9: Patient Health Questionnaire-9
DSM-IV: Diagnostic and statistical manual of mental disorders-IV
MDD: Major depressive disorder
PHQ-2: Patient Health Questionnaire-2
SD: Standard deviation
GAD-7: Generalized anxiety disorder scale-7
IAT: Internet addiction test
CD-RISC-10: Connor-Davidson resilience Scale-10
PYD-VSF: 5Cs positive youth development scale-very short form
GGM: Gaussian graphical model
EBICglasso: Extended Bayesian information criterion graphical least absolute shrinkage and selection operator
ROC: Receiver operating characteristic
AUC: Area under the curve
PHQ-2 N: New Patient Health Questionnaire-2
