# Supplementary material for: A new PHQ-2 for Chinese adolescents: identifying core items of the PHQ-9 by network analysis
Source: Child Adolesc Psychiatry Ment Health. 2023 Jan 21;17:11. doi: 10.1186/s13034-023-00559-1 (PMC9862239; doi:10.1186/s13034-023-00559-1)
Supplement: Supplementary file 1 — Additional file 1: Figure S1. Network structure of PHQ-9 items. Note. The stronger the association between nodes, the thicker and more saturated the edge is represented in the network. Blue edges represent positive associations. Figure S2. Accuracy of edge weights. Note. The gray area shows the bootstrapped confidence intervals of the estimated edge weights for the estimated network. The red values (connected by the red line) indicate the sample mean values for the bootstrapped edge weights. The black values indicate the estimated edge weights. Figure S3. Stability of nose strength centrality. Note. The plot shows the average correlation between the strength for the estimated network and the bootstrapped network. The lines indicates the mean correlation between centrality measures and the area around the indicates the 2.5th till the 97.5th quartile. Table S1. Normative data of the PHQ-9. [file 13034_2023_559_MOESM1_ESM.docx]

**Additional file**

| 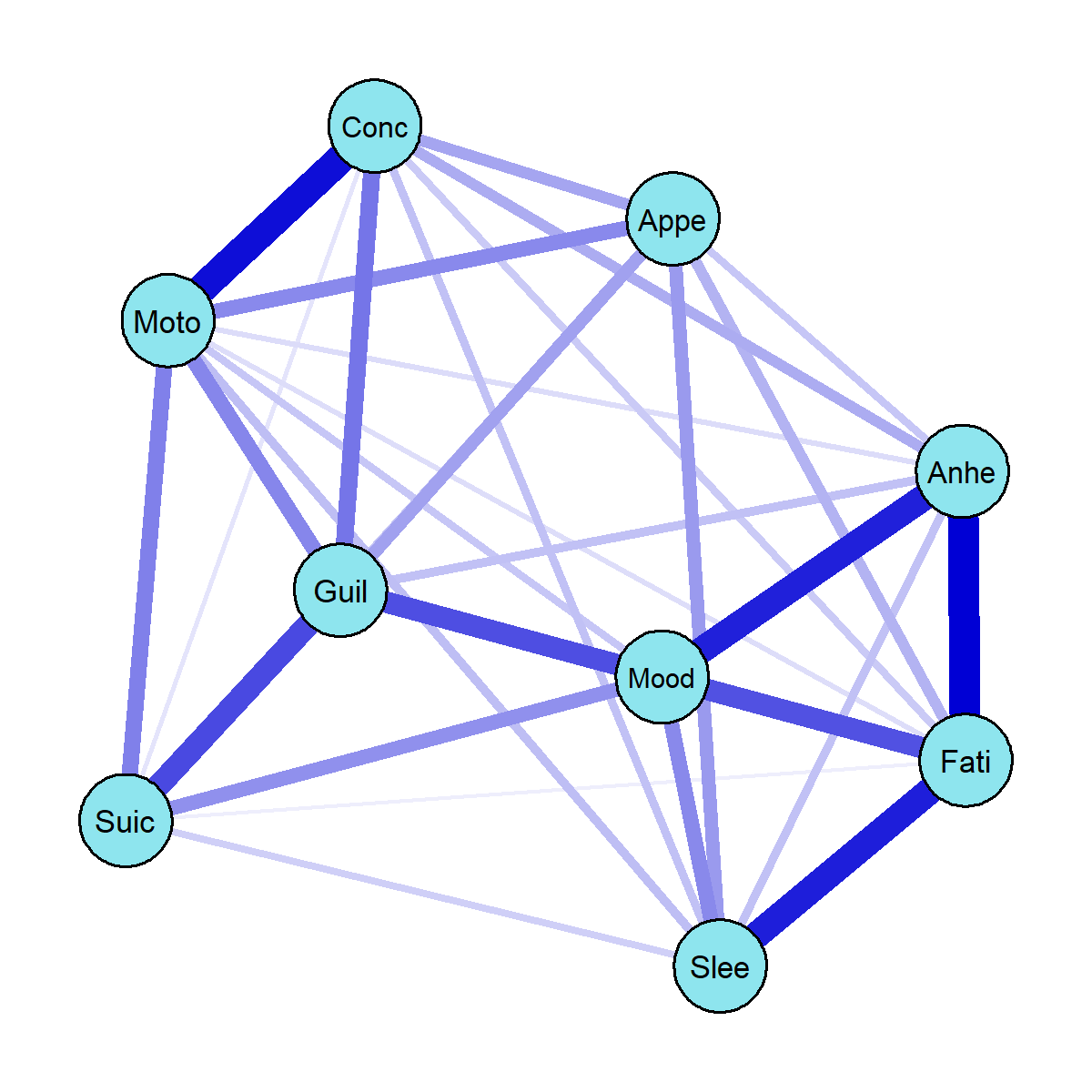  (a) Sample 1-males | 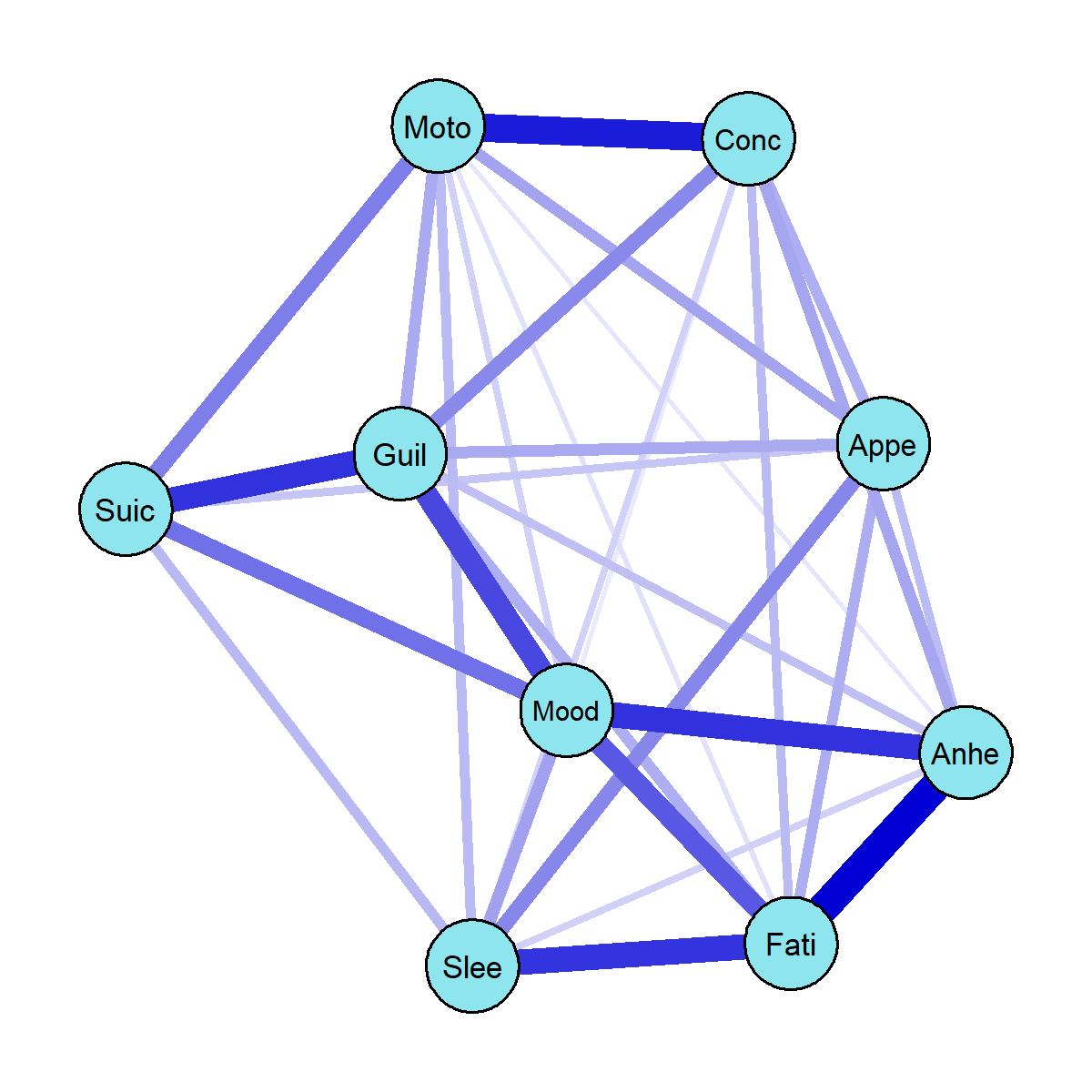  (b) Sample 1-females |
| --- | --- |
| 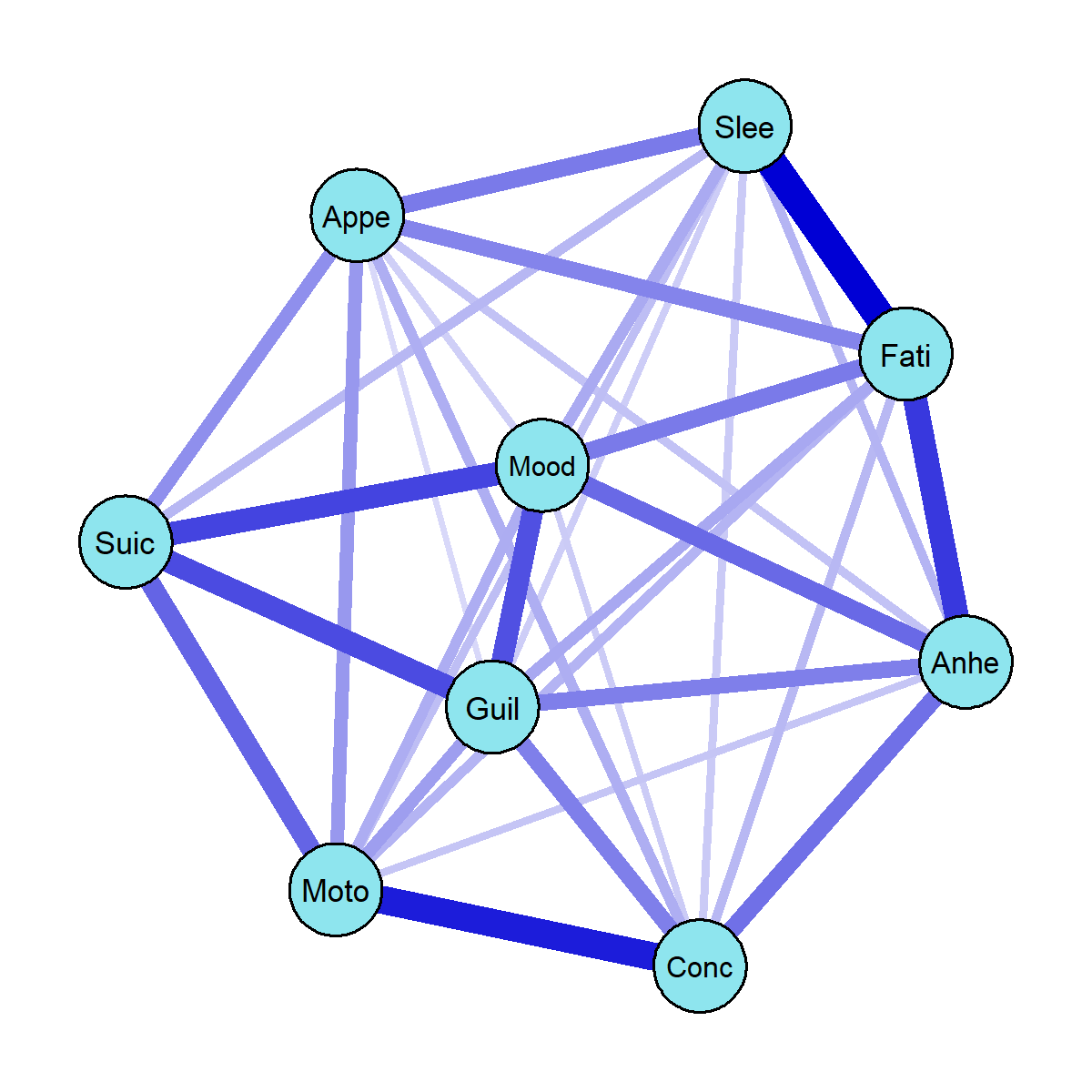  (c) Sample 2-males | 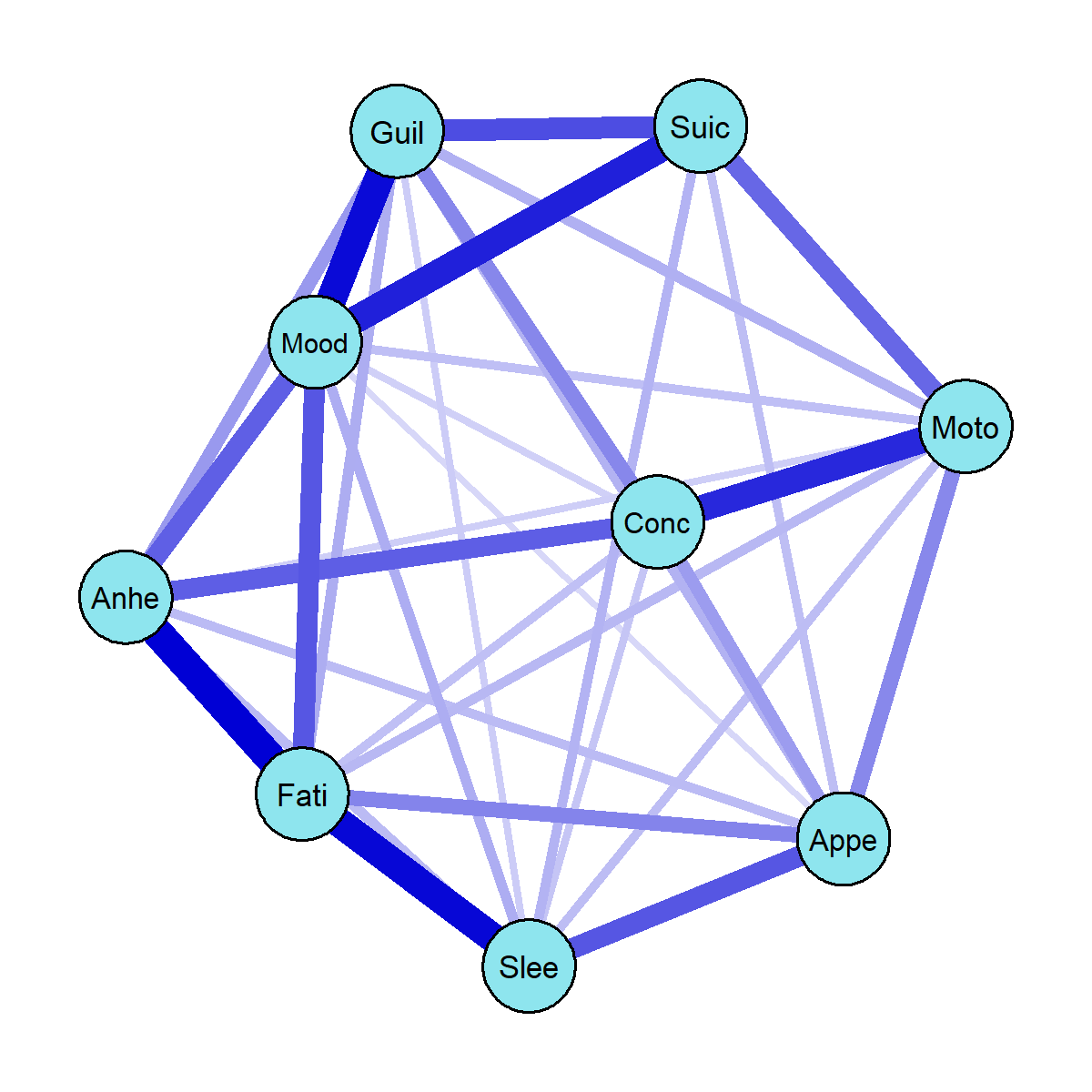  (d) Females 2-females |

**Figure S1.** Network structure of PHQ-9 items

Note. The stronger the association between nodes, the thicker and more saturated the edge is represented in the network. Blue edges represent positive associations.

| 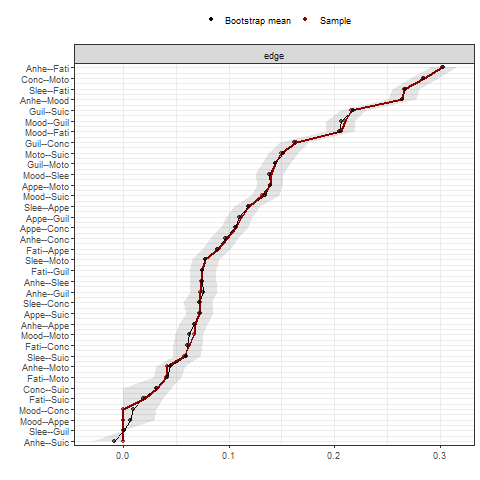  (a) Sample 1-males | 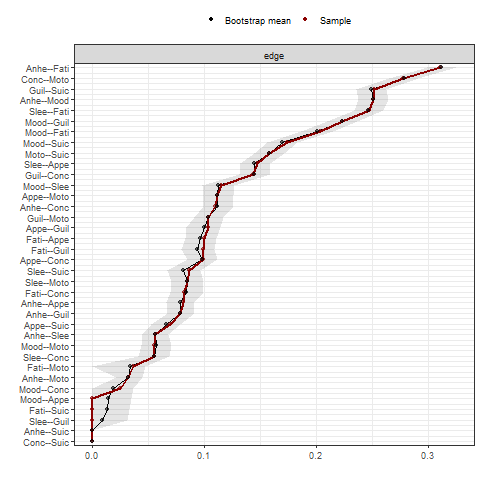(b) Sample 1-females |
| --- | --- |
| 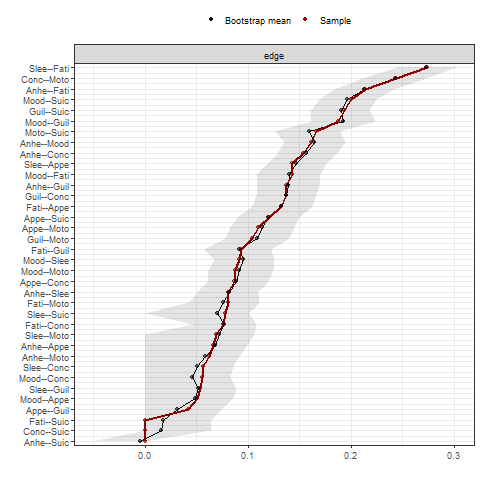  (c) Sample 2-males | 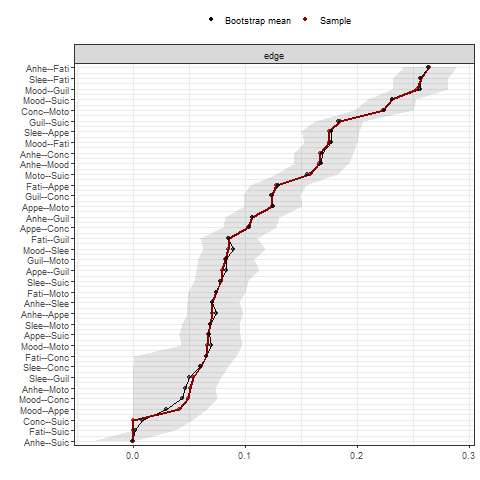(d) Females 2-females |

**Figure S2. Accuracy of edge weights**

Note. The gray area shows the bootstrapped confidence intervals of the estimated edge weights for the estimated network. The red values (connected by the red line) indicate the sample mean values for the bootstrapped edge weights. The black values indicate the estimated edge weights.

| 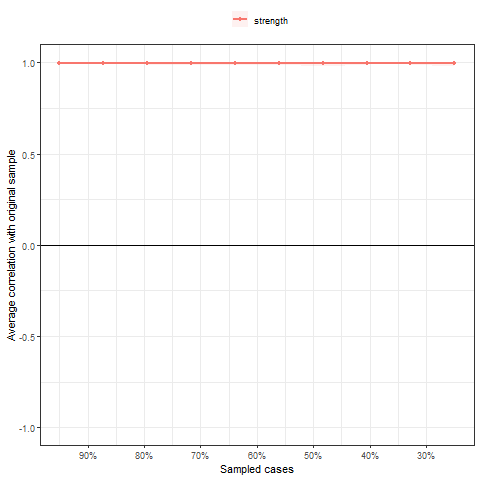  (a) Sample 1-males | 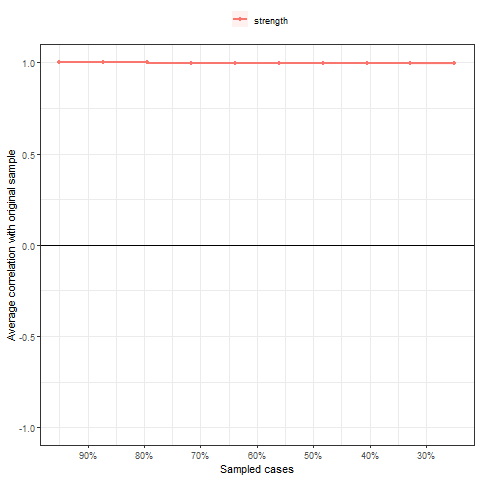(b) Sample 1-females |
| --- | --- |
| 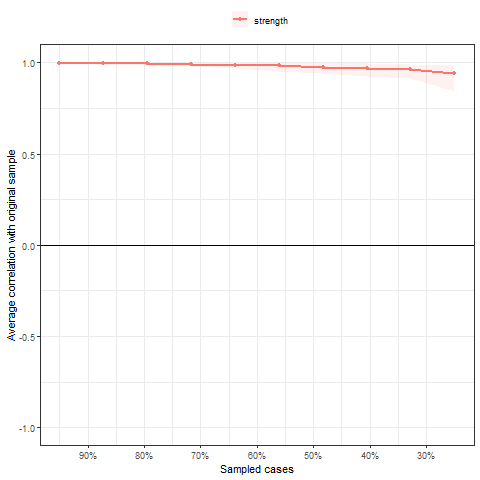  (c) Sample 2-males | 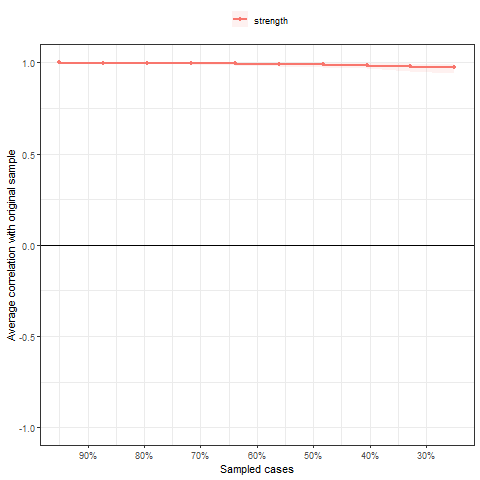(d) Females 2-females |

**Figure S3. Stability of nose strength centrality**

Note. The plot shows the average correlation between the strength for the estimated network and the bootstrapped network. The lines indicates the mean correlation between centrality measures and the area around the indicates the 2.5th till the 97.5th quartile.

**Table S1. Normative data of the PHQ-9**

| Total score | Male | | Female | |
| --- | --- | --- | --- | --- |
|  | Percentage | Cumulative percentage | Percentage | Cumulative percentage |
| 0 | 30.4 | 30.4 | 21.4 | 21.4 |
| 1 | 9.6 | 40.0 | 8.5 | 29.9 |
| 2 | 7.9 | 48.0 | 7.6 | 37.5 |
| 3 | 7.1 | 55.1 | 7.3 | 44.7 |
| 4 | 6.8 | 61.9 | 7.4 | 52.1 |
| 5 | 5.8 | 67.7 | 6.4 | 58.5 |
| 6 | 4.9 | 72.5 | 6.0 | 64.5 |
| 7 | 4.4 | 76.9 | 5.7 | 70.1 |
| 8 | 4.8 | 81.7 | 5.4 | 75.5 |
| 9 | 5.6 | 87.2 | 5.3 | 80.8 |
| 10 | 2.2 | 89.4 | 3.0 | 83.8 |
| 11 | 1.7 | 91.2 | 2.6 | 86.4 |
| 12 | 1.5 | 92.7 | 2.0 | 88.4 |
| 13 | 1.2 | 93.9 | 1.9 | 90.3 |
| 14 | 1.0 | 94.9 | 1.5 | 91.9 |
| 15 | 0.8 | 95.7 | 1.4 | 93.2 |
| 16 | 0.7 | 96.4 | 1.0 | 94.3 |
| 17 | 0.5 | 97.0 | 1.0 | 95.3 |
| 18 | 1.0 | 97.9 | 1.1 | 96.3 |
| 19 | 0.3 | 98.3 | 0.6 | 97.0 |
| 20 | 0.2 | 98.5 | 0.5 | 97.5 |
| 21 | 0.2 | 98.7 | 0.5 | 98.0 |
| 22 | 0.2 | 98.9 | 0.4 | 98.4 |
| 23 | 0.2 | 99.1 | 0.3 | 98.7 |
| 24 | 0.2 | 99.3 | 0.3 | 99.0 |
| 25 | 0.2 | 99.4 | 0.3 | 99.3 |
| 26 | 0.1 | 99.5 | 0.2 | 99.5 |
| 27 | 0.5 | 100.0 | 0.5 | 100.0 |
